# Editorial: Unraveling the molecular mechanisms of cytokine signaling in regulating inflammatory diseases

**DOI:** 10.3389/fimmu.2025.1563469

**Published:** 2025-02-11

**Authors:** Jian Zheng, Huawei Mao, Wai Po Chong

**Affiliations:** ^1^ Department of Microbiology and Immunology, University of Louisville, Louisville, KY, United States; ^2^ Center for Predictive Medicine, University of Louisville, Louisville, KY, United States; ^3^ Department of Immunology, Ministry of Education Key Laboratory of Major Diseases in Children, Beijing Children’s Hospital, Capital Medical University, Beijing, China; ^4^ National Center for Children’s Health, Beijing Key Laboratory for Genetics of Birth Defects, Beijing, China; ^5^ Department of Nephrology and Rheumatology, Children’s Hospital of Xinjiang Uygur Autonomous Region, Xinjiang Hospital of Beijing Children’s Hospital, Xinjiang, China; ^6^ School of Chinese Medicine, Hong Kong Baptist University, Hong Kong, Hong Kong SAR, China; ^7^ Institute for Research and Continuing Education, Hong Kong Baptist University, Shenzhen, China

**Keywords:** cytokin, inflammation, autoimmunity, infection - immunology, signaling pathway

## 1 Background

Chronic and dysregulated inflammation is a hallmark of many autoimmune and inflammatory diseases, significantly impacting patient health and quality of life. Despite their prevalence, the intricate molecular mechanisms underlying cytokine regulation in these conditions remain poorly understood. This Research Topic aims to bridge this knowledge gap by exploring the complex network of cytokine signaling pathways and their role in immune regulation during inflammatory diseases. Through a collection of nine research articles and six review articles, we delve into the latest discoveries and insights, providing a comprehensive overview of the current state of research in this critical area.

## 2 Cytokine regulation in infection and associated inflammatory responses

Cambon et al. investigated cytokine profiles in the lung compartment of COVID-19 patients, particularly those with acute respiratory distress syndrome (C-ARDS). The authors evaluated caspase-1 activation, IL-1 signature, and other inflammatory cytokine pathways using post-mortem lung tissue, bronchoalveolar lavage fluid (BALF), and serum. Their findings revealed elevated levels of proinflammatory molecules such as caspase-1, IL-1β, IL-1Ra, IL-6, IFN-γ, and CXCL-10 in BALF from steroid-treated C-ARDS patients, highlighting the predominant IL-1β/IL-6 signature and IFN-γ-associated chemokines despite steroid treatment. This study underscores the potential of targeting these pathways to improve treatment response and limit lung damage in ARDS.

In another study, Vorobyeva et al. developed an ex vivo model of SARS-CoV-2 lung infection to study cytokine production. Their findings revealed elevated concentrations of proinflammatory mediators, namely G-CSF, GM-CSF, GRO-α, IFN-γ, IL-6, IL-8, IP-10, MCP-3, MIP-1α, PDGF-AA, and VEGF in infected lung tissue, reflecting the cytokine alterations observed in COVID-19 patients. This model provides a valuable platform to investigate the mechanisms of SARS-CoV-2 infection and to test antiviral drugs.

The study by Bédard-Matteau J. et al. identified IL-17F as a key cytokine associated with severe COVID-19. Elevated IL-17F levels were found in severe cases, promoting neutrophil adhesion to endothelial cells via ERK1/2 and p38 MAPK-dependent pathways. These findings highlight the potential of targeting IL-17F signaling to mitigate neutrophilic inflammation and immunothrombosis in severe COVID-19.

Von Stemann et al. examined the association of cytokine autoantibodies (c-aAbs) with community-acquired pneumonia (CAP). They measured c-aAbs targeting various cytokines in plasma samples from 665 CAP patients. The results indicated that high-titer type 1 IFN c-aAb is more prevalent in men with SARS-CoV-2 infection, while GM-CSF c-aAb is associated with asthma and bronchiectasis comorbidities in men. These findings suggest that c-aAb specificity, comorbidity, and sex influence clinical outcomes in CAP, providing insights for personalized treatment strategies.

## 3 Cytokine signaling in autoimmune and inflammatory conditions

The study by Yang et al. investigated the therapeutic potential of myeloid-derived growth factor (MYDGF) in primary Sjögren’s syndrome (pSS). Using a mouse model, the authors demonstrated that MYDGF treatment alleviates pSS symptoms by increasing salivary flow rate, reducing lymphocyte infiltration, and promoting M2 macrophage polarization. The study identifies the suppression of the CX3CL1/CX3CR1 axis as a key mechanism, suggesting MYDGF as a promising therapeutic target for pSS.

Lee et al. explored the role of the pregnane X receptor (PXR) in particulate matter (PM)-induced inflammation in atopic dermatitis (AD). Their findings indicated that PXR activation reduces type 17 inflammation by inhibiting the NF-κB pathway, suggesting PXR as a therapeutic target for controlling PM-induced AD aggravation. In addition, Xu et al. review the emerging role of protease-activated receptor 2 (PAR2) in various skin conditions, such as atopic dermatitis, psoriasis, vitiligo, and melasma. The review highlights the involvement of PAR2 in the cutaneous microenvironment and associated comorbidities, proposing it as a key target for therapeutic intervention.

Ouyang and Dai employed Mendelian randomization to explore the causal relationships between systemic inflammatory cytokines and adhesive capsulitis (AC). Their findings established causal associations between IP-10, RANTES, SDF-1α, TNF-α levels, and AC risk, offering new avenues for understanding AC pathogenesis and developing clinical management strategies.

Liu et al. identified MCP-3 as a significant prognostic biomarker for severe fever with thrombocytopenia syndrome (SFTS). Elevated MCP-3 levels were found to correlate with adverse outcomes. These findings provide a valuable tool for predicting prognosis and understanding the cytokine-mediated pathogenesis of SFTS.

The bibliometric review by Liu et. al. focused on leukocyte cell-derived chemotaxin-2 (LECT2). The study identified liver diseases, systemic inflammatory diseases, and amyloidosis as current research focuses, highlighting the potential of LECT2 for clinical diagnosis and treatment.

In another review, Guo et al. explored the role of the CCL2/CCR2 signaling axis in inflammatory and fibrotic diseases. CCL2, a key cytokine, binds to its receptor CCR2, modulating the recruitment and activation of immune cells and influencing the progression of fibrosis in various organs. The paper highlights recent advances in diagnosing and treating fibrotic diseases linked to this pathway and calls for further research to elucidate its clinical significance in different organ systems.

## 4 Advances in asthma immunology

In their review, Xie et al. discussed recent advances in asthma immunology, emphasizing the heterogeneity of immune processes and phenotypes. The paper explored the key cellular and molecular mediators involved in type 2-high and type 2-low asthma endotypes and reviews innovative biological and targeted therapies. Understanding the dynamic and complex immunopathology of asthma is crucial for the development of personalized interventions.

## 5 Regulatory mechanisms of cytokine signaling

Chen et al. investigated the role of LTβR signaling in chemotherapy-induced mucosal damage. The authors’ suggested that LIGHT produced by T cells activates LTβR-RelB signaling in intestinal epithelial cells, promoting mucosal repair and offering insights into therapeutic strategies for chemotherapy-induced damage.

The review by Zong et al. explored the cytokine signaling pathways that regulate Treg cells and their implications for autoimmune diseases, transplant rejection, and cancer. Understanding these pathways offers potential for the development of Treg-based immunotherapies to restore immune balance.

Huang et al. examined the bidirectional regulation of the TRPM2 channel in oxidative stress, inflammation, and ischemia-reperfusion (I/R) injury. The role of the TRPM2 channel in exacerbating or protecting against cellular damage under different conditions provides insights into potential therapeutic strategies for related diseases.

## 6 Conclusion

This Research Topic highlights the critical role of cytokine regulation in chronic inflammatory and autoimmune diseases. By unraveling the molecular mechanisms underlying cytokine signaling pathways, we gain valuable insights into disease progression and identify potential therapeutic targets. This research contributes to the advancement of precision medicine and the development of novel treatments, ultimately improving patient outcomes in autoimmune and inflammatory diseases.

